# Simultaneous assessment of cardiac metabolism and perfusion using copolarized [1‐^13^C]pyruvate and ^13^C‐urea

**DOI:** 10.1002/mrm.26106

**Published:** 2016-01-07

**Authors:** Angus Z. Lau, Jack J. Miller, Matthew D. Robson, Damian J. Tyler

**Affiliations:** ^1^ Division of Cardiovascular Medicine, Radcliffe Department of MedicineUniversity of OxfordOxfordUnited Kingdom; ^2^ Department of Physiology, Anatomy, and GeneticsUniversity of OxfordOxfordUnited Kingdom; ^3^ Department of Physics, Clarendon LaboratoryUniversity of OxfordOxfordUnited Kingdom

**Keywords:** hyperpolarization, metabolism, first‐pass perfusion, cardiac, ^13^C

## Abstract

**Purpose:**

To demonstrate the feasibility of imaging a bolus of co‐polarized [1‐^13^C]pyruvate and ^13^C‐urea to simultaneously assess both metabolism and perfusion in the rodent heart.

**Methods:**

Copolarized [1‐^13^C]pyruvate and ^13^C‐urea was imaged using a multi‐echo, flow‐sensitized spiral pulse sequence. Healthy rats were scanned in a two‐factor factorial design (n = 12 total; metabolism: overnight fasting versus fed with dichloroacetate injection; perfusion: rest versus adenosine stress‐induced hyperemia).

**Results:**

Alterations in metabolism were detected by changes in pyruvate metabolism into ^13^C‐bicarbonate. Statistically independent alterations in perfusion were detected by changes in myocardial pyruvate and urea signals.

**Conclusion:**

The new pulse sequence was used to obtain maps of metabolism and perfusion in the rodent heart in a single acquisition. This hyperpolarized ^13^C imaging test is expected to enable new studies in which the cardiac metabolism/perfusion mismatch can be studied in the acute environment. Magn Reson Med 77:151–158, 2017. © 2016 The Authors Magnetic Resonance in Medicine published by Wiley Periodicals, Inc. on behalf of International Society for Magnetic Resonance in Medicine

## INTRODUCTION

Following acute myocardial infarction, the assessment of tissue viability and the presence of the salvageable “area at risk” must be performed rapidly 1. In particular, the restoration of blood flow to the ischemic heart restores normal function and improves clinical outcomes in patients with viable tissue; however, this results in unnecessary costs and clinical risk in patients with nonviable tissue 2. The identification of hibernating myocardium, which is characterized by reduced myocardial perfusion, impaired contractile function, and intact metabolism, can be used to guide the clinical decision to proceed with revascularization 3.

Recent developments in hyperpolarized magnetic resonance using the dissolution dynamic nuclear polarization method have enabled transient signal increases in ^13^C‐labeled molecules relative to thermal equilibrium in excess of 10,000‐fold 4. This molecular imaging modality enables imaging of in vivo metabolic changes in real time, within the initial minute of injection into the body. For example, injection of hyperpolarized [1‐^13^C]pyruvate provides key biochemical information regarding flux into the tricarboxylic acid cycle via observation of ^13^C‐bicarbonate as well as anaerobic metabolism via observation of ^13^C‐labeled lactate 5, 6, 7. Injection of metabolically inert compounds, such as ^13^C‐urea, provides a directly quantifiable probe of tissue perfusion, due to the low signal background of the images, and the linear signal response with respect to concentration 8, 9. Thus, these two hyperpolarized ^13^C measurements of tissue metabolism and perfusion have great potential in assessing tissue viability and the presence of hibernating myocardium.

Nevertheless, the use of hyperpolarized ^13^C substrates to identify viable myocardium and guide clinical decision‐making remains infeasible, due to the need for two separate injections of ^13^C‐pyruvate and ^13^C‐urea. Simultaneous hyperpolarization (ie, copolarization) of multiple ^13^C‐labeled substrates has been reported and offers an attractive method to obtain multiple readouts of both enzymatic and physiological properties in vivo in a single imaging experiment 10, 11, 12. This approach has not been explored in the context of cardiac imaging. Recent work using flow‐encoding gradients has overcome the strict spatial resolution requirements in small animal cardiac imaging by nulling signal from rapidly moving intravascular ^13^C‐compounds 13, 14.

In this study, we investigated the feasibility of using an injection of copolarized [1‐^13^C]pyruvate and ^13^C‐urea combined with a flow‐sensitized spiral multiecho pulse sequence to simultaneously assess both myocardial metabolism and perfusion in a single acquisition. The approach is evaluated in the healthy in vivo rat heart in which metabolism can be modulated by fasting, and perfusion can be independently modulated by infusion of the coronary vasodilator adenosine. We demonstrate the sensitivity of the new method to detect changes in both of these physiological parameters following a single injection of hyperpolarized ^13^C substrates. This is an important development that will enable rapid identification of viable tissue following acute myocardial infarction, which is characterized by reduced myocardial perfusion alongside intact metabolism.

## METHODS

All experiments were performed on an Agilent 7 T MRI system (Agilent, Santa Clara, California, USA). All animal investigations conformed to the Home Office Guidance on the Operation of the Animals (Scientific Procedures) Act of 1986 in accordance with institutional guidelines and were approved by the University of Oxford Animal Ethics Review Committee.

### Pulse Sequence

**Figure 1:**
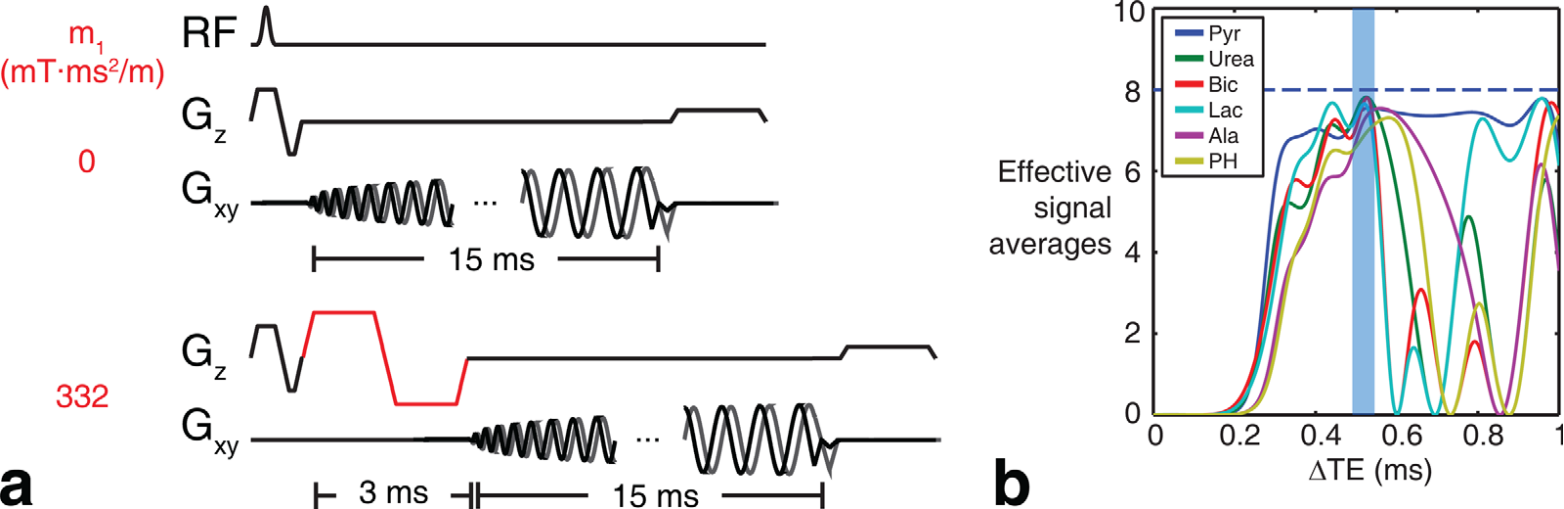
Flow sensitized ECG‐gated spiral sequence for combined first‐pass perfusion imaging and metabolic imaging of ^13^C in the heart. (**a**) The bipolar flow sensitizing gradient (red) is toggled in alternate TRs, dephasing signal from within the cardiac chambers. A total of nine TRs (8 TEs + 1 FID) is used to resolve the ^13^C metabolites. (**b**) The echo spacing (ΔTE) is chosen by maximizing the effective NSA, shown for each metabolite. The blue bar indicates the range of ΔTE for which the minimum NSA over all species is above 80% of the maximum (dashed line; NSA_max_ = 8 echoes).

Figure 1a shows the multiecho electrocardiogram (ECG)‐gated spiral sequence used to obtain axial dynamic ^13^C images in the heart. A single‐shot 15‐ms spiral readout is used for spatial encoding. The echo time is incremented between repetition times (TRs), and a total of eight echoes and one nonselective spectrum are acquired per block. The echo times (TEs) were chosen to maximize the number of signal averages (NSA) over all chemical species, in the IDEAL reconstruction (Figure 1b) 15, 16. Given an encoding matrix *E*_*n.m*_ = exp(*iω*_*n*_TE_*m*_), where *ω*_*n*_ represents the frequencies for each metabolite, the NSA metric describes the noise performance for each species as the inverse of each diagonal component of the covariance matrix of *E, NSA*_*n*_ = 1/((*E^T^E*)^−1^)_*n*,*n*_. In a noise‐free reconstruction, NSA becomes equal to the number of echoes. In this study, an echo time increment ΔTE = 0.5 ms was chosen so that the minimum NSA over all chemical species was at least 80% of the number of echoes 8 used.

A bipolar flow sensitizing gradient, characterized by flow encoding moment m_1_, was inserted after the slice‐selective excitation and before the start of the readout to suppress flowing spins in the cardiac chambers via intravoxel dephasing, as described previously 14. The full sequence was operated by acquiring all echoes in a non–flow‐sensitized block (m_1_ = 0 mT · ms^2^/m), followed by all echoes in a flow‐sensitized block (m_1_ = 332 mT · ms^2^/m), and then repeating this schedule for 2 min. Given a typical rodent heart rate of 360 bpm, this schedule yields a total temporal resolution of 3 s for two sets of ^13^C metabolite images.

### Animal Handling

Male Wistar rats (n = 12, weight = 390 ± 70 g) were scanned in this study. Anesthesia was induced at 2.5%–3% isoflurane in oxygen and nitrous oxide (4:1, total of 2 L/min). Anesthesia was maintained by means of 2% isoflurane delivered to, and scavenged from, a nose cone during the experiment. A tail vein catheter was placed for intravenous injection of the hyperpolarized agents, ^13^C‐urea and [1‐^13^C]pyruvate. A separate tail vein catheter was placed in the contralateral tail vein for delivery of adenosine during the stress‐induced hyperemia experiments, described below. Animals were then placed in a home‐built animal handling system 17. Body temperature was maintained using air heating, and a two‐lead ECG for cardiac gating was recorded using leads placed subcutaneously into the upper forelimbs.

### Metabolic and Perfusion Modulation

To assess the sensitivity of the copolarization approach to changes in both metabolism and perfusion, the animals were split into 4 groups (n = 3 each) in a 2 × 2 factorial design. Cardiac metabolism was altered by either overnight fasting, or placing them in the fed state along with intravenous injection of a bolus of the pyruvate dehydrogenase kinase (PDK) inhibitor, dichloroacetate (DCA; 30 mg/kg, neutralized to pH 7) through the primary tail vein catheter 10 min prior to hyperpolarized ^13^C injection. Dichloroacetate prevents PDK‐mediated inhibition of pyruvate dehydrogenase (PDH), which has the effect of increasing PDH flux and thus production of ^13^C‐bicarbonate from hyperpolarized [1‐^13^C]pyruvate 18, 19. Cardiac perfusion was altered as described previously via continuous infusion of adenosine, a coronary vasodilator (concentration, 3 mg/mL; flow rate, 280 μg/kg/min) into a separate tail vein using a small animal infusion pump (Harvard Apparatus, Holliston, Massachusetts, USA). The adenosine infusion was maintained throughout the ^13^C scan. No change in heart rate was detected during adenosine stress (mean heart rate immediately prior to infusion 390 ± 30 bpm at rest, 360 ± 40 bpm during stress). Animals were monitored during and after adenosine stress for signs of atrial fibrillation or other adverse effects.

### Hyperpolarization

A sample for copolarization was prepared by first freezing a layer of ^13^C‐urea (6.4 M with 15 mM OX063 trityl radical and 0.7 mM Gd‐DOTA (Dotarem, Guerbet, France) in a 6:4 w/w glycerol/water solution) in a liquid nitrogen bath at the bottom of the sample holder. A second layer of [1‐^13^C]pyruvic acid (14 M neat with 15 mM OX063 trityl radical and 0.8 mM Gd‐DOTA) was added on top to prevent mixing. Polarization was performed using a prototype dynamic nuclear polarization hyperpolarizer at 93.979 GHz and 100 mW for 120 min 4. The microwave frequency was chosen to maximize the [1‐^13^C]pyruvate polarization build‐up in order to maximize the signal‐to‐noise ratio of the downstream metabolites of [1‐^13^C]pyruvate, which are expected to have the lowest signal. Dissolution was performed at a pressure of 10 bar and a temperature of ∼170 °C in 6 mL of NaOH solution resulting in a solution of approximately pH 7 containing injected substrate concentrations of 80 mM (pyruvate) and 64 mM (urea), corresponding to dose levels used in previous studies of cardiac metabolism 6 and perfusion 14. The hyperpolarized solution was transferred from the polarizer into a previously described low‐field (2–10 mT) magnetic holder, and the solution was transferred to the fringe field (2 mT) of the magnet prior to injection 14. Two mL of copolarized ^13^C‐urea/[1‐^13^C]pyruvate was injected over 20 s via the tail vein. This injection timing was chosen to maximize the number of frames during which pyruvate and urea were visible in the heart, according to previous studies of cardiac perfusion using hyperpolarized ^13^C‐urea 14.

### MRI Acquisitions

^1^H images were acquired using a 72‐mm inner diameter dual‐tuned birdcage transmit/receive ^1^H/^13^C coil (Rapid Biomedical GmbH, Rimpar, Germany). ^13^C images were acquired using the same volumetric coil for radiofrequency (RF) transmission, and a two‐channel surface receive array (two 2 × 4 cm^2^ elements oriented in the left–right direction) for signal reception (Rapid Biomedical GmbH, Rimpar, Germany). A volume covering a 70‐mm slab including the heart was used for shimming using a three‐dimensional gradient echo automated shim routine 20. A prospectively triggered segmented cine FLASH sequence was used to assess cardiac function in a transverse midventricular slice using the following parameters: TE = 1.36 ms; TR = 4.6 ms; field of view = 80 × 80 mm^2^; acquired in‐plane resolution = 0.8 × 0.8 mm^2^; slice thickness = 5 mm; and flip angle = 20 °.

Images of [1‐^13^C]pyruvate, its metabolites, and ^13^C‐urea through the heart were obtained using the aforementioned ECG‐gated single‐shot spiral sequence. For each imaging block, eight echoes and one free induction decay (FID) were acquired using the following parameters: TR = 1 RR interval; flip angle = 10 °; field of view = 70 × 70 mm^2^; matrix = 64 × 64; acquired in‐plane resolution = 1.75 × 1.75 mm^2^; slice thickness = 5 mm; excitation pulse duration = 0.5 ms; and readout duration = 15 ms. The readout duration for the FID acquisition was 64 ms, giving a spectral resolution of 15 Hz (0.2 ppm at 7 T). For the non–flow‐sensitized images, the initial echo time was chosen to be TE_0_ = 0.8 ms, with ΔTE = 0.5 ms, to minimize $T_{2}^{*}$ related signal dephasing. For the flow‐sensitized images, a 3‐ms bipolar gradient (m_1_ = 332 mT · ms^2^/m) was inserted between slice‐selective excitation and the spiral readout, yielding an initial echo time of TE_0_ = 4.3 ms.

### Image Reconstruction

**Figure 2:**
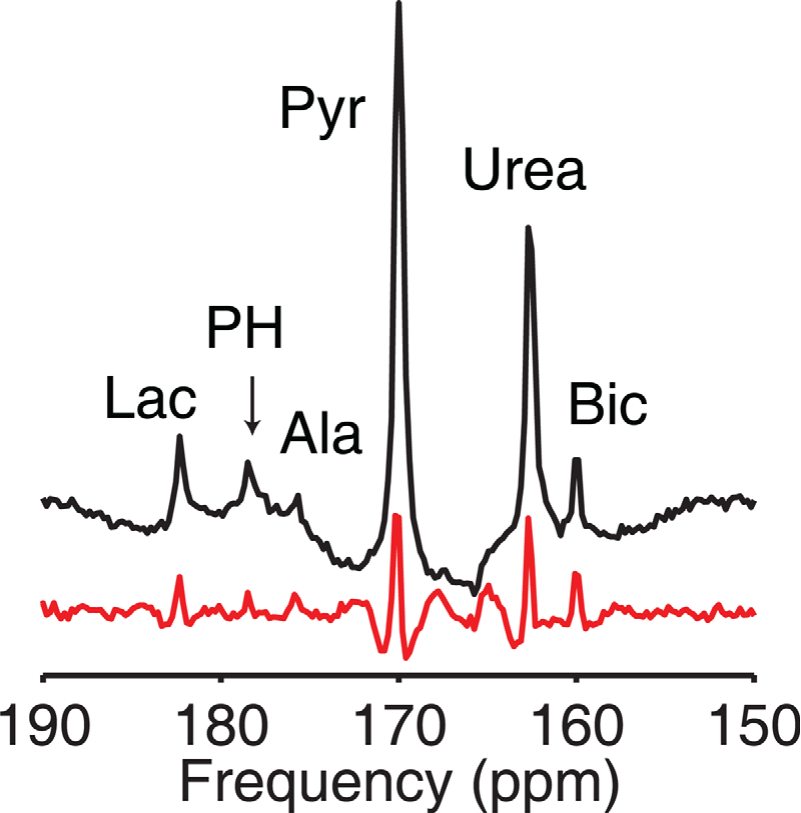
Summed real spectra obtained without (black) and with (red) flow encoding, in the fed state with resting perfusion levels. The intravascular pyruvate and urea lines are typically reduced in amplitude, whereas intracellular bicarbonate remains similar in amplitude.

The FID obtained during each imaging block was used to identify the relevant ^13^C metabolites. Following injection of copolarized [1‐^13^C]pyruvate and ^13^C‐urea, a total of six ^13^C resonances appear (Fig. 2), corresponding to ^13^C‐lactate (182.2 ppm), ^13^C‐pyruvate hydrate (178.2 ppm), ^13^C‐alanine (175.6 ppm), ^13^C‐pyruvate (170 ppm), ^13^C‐urea (162.5 ppm), and ^13^C bicarbonate (160 ppm). The spectral components in the FIDs were fitted using the AMARES algorithm to obtain single‐slice time courses for each ^13^C metabolite for both non–flow‐sensitized and flow‐sensitized imaging blocks 21. Using the measured frequencies for each spectral line, the encoding matrix *E*_*n.m*_ = exp(*iω*_*n*_TE_*m*_) was constructed, and the pseudo‐inverse was applied to each spatial k‐space point in the multiecho dataset 16, 22. In this reconstruction, the B_0_ is assumed to be homogeneous across the heart, and the center frequency was set by identifying the pyruvate frequency in each frame.

The single‐shot spiral trajectory was predicted using a premeasured gradient impulse response function 23. The k‐space samples were filtered in the time domain using a 30‐Hz exponential line broadening function, yielding a final reconstructed in‐plane resolution of 5 × 5 mm^2^. The non‐Cartesian k‐space samples were gridded using the nonuniform fast Fourier transform 24.

### Data Analysis

The heart was segmented manually into right ventricle, left ventricle (LV), and myocardial tissue using the ^1^H cine images. The magnitude images for each ^13^C metabolite were used for data analysis. The signal means within each compartment were baseline corrected using the mean pyruvate LV signal prior to appearance of the hyperpolarized agents. The metabolites derived from [1‐^13^C]pyruvate were normalized to the maximum non–flow‐suppressed pyruvate signal within the LV. Similarly, the ^13^C‐urea signals were normalized to the maximum non–flow‐suppressed urea signal within the LV. This normalization procedure is intended to remove any interstudy differences in coil positioning and substrate polarization. The metabolite images (bicarbonate and lactate) were set to zero if the corresponding spectral amplitude in that frame fell below 2% of the maximum pyruvate signal to avoid artifacts in the multiecho reconstruction caused by the large urea and pyruvate peaks.

Areas under the curve (AUC) were then calculated for each compartment. As described previously, a measure of myocardial perfusion was taken to be the ratio between the flow‐suppressed tissue AUC and non–flow‐suppressed LV AUC for both pyruvate and urea. A measure of metabolic activity was taken to be the ratio between flow‐suppressed tissue bicarbonate or lactate AUC and the non–flow‐suppressed pyruvate LV AUC. Two‐way analysis of variance (ANOVA) with Sidak's multiple comparisons correction was used to compare these measures across the four groups. Statistical significance was considered at *P* < 0.05.

## RESULTS

### In Vivo Study

Figure 2 shows a sample in vivo slice‐selective cardiac spectrum obtained following injection of copolarized ^13^C pyruvate and urea in the fed state with resting perfusion. The additional first‐order phase in the flow‐sensitized spectrum arose from the increased TE (0.8 versus 4.3 ms) due to the insertion of the bipolar gradient. In this group, the flow‐sensitized spectrum showed reduced amplitude pyruvate (15%) and urea resonances (20% of the non–flow‐suppressed amplitude). ^13^C‐bicarbonate, which is produced in the mitochondria via PDH, is largely intracellular, and thus remained similar in amplitude in both spectra (80% of the non–flow‐suppressed amplitude). The residual signal decrease was consistent with an echo time difference of 3.5 ms and fitted line widths of 15–20 Hz. ^13^C‐lactate, which is produced via lactate dehydrogenase–mediated exchange in the myocardium as well as in the blood, showed a similar signal decrease (70% of the non–flow‐suppressed amplitude).

**Figure 3:**
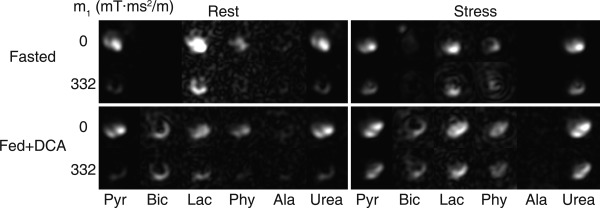
In vivo data showing hyperpolarized pyruvate, its downstream metabolites (bicarbonate, lactate, pyruvate hydrate, and alanine), and urea in the rat heart. Each set of images corresponds to one combination of perfusion and metabolic state. Images are shown either with no flow encoding (top row) or with flow sensitization (bottom row). The images are cropped to a 27 × 27 mm^2^ field of view.

Figure 3 shows representative cardiac images of hyperpolarized ^13^C pyruvate, its metabolites, and ^13^C‐urea, in the four combinations of metabolic and perfusion states. The images are summed over the entire acquisition time. Infusion of adenosine led to vasodilation of the tissue and an increase in myocardial pyruvate and urea signal, as shown by the flow‐suppressed images in each of the frames. Modulation of the metabolic state of the tissue by feeding and a bolus injection of DCA resulted in increased myocardial bicarbonate signal relative to fasted tissue.

**Figure 4:**
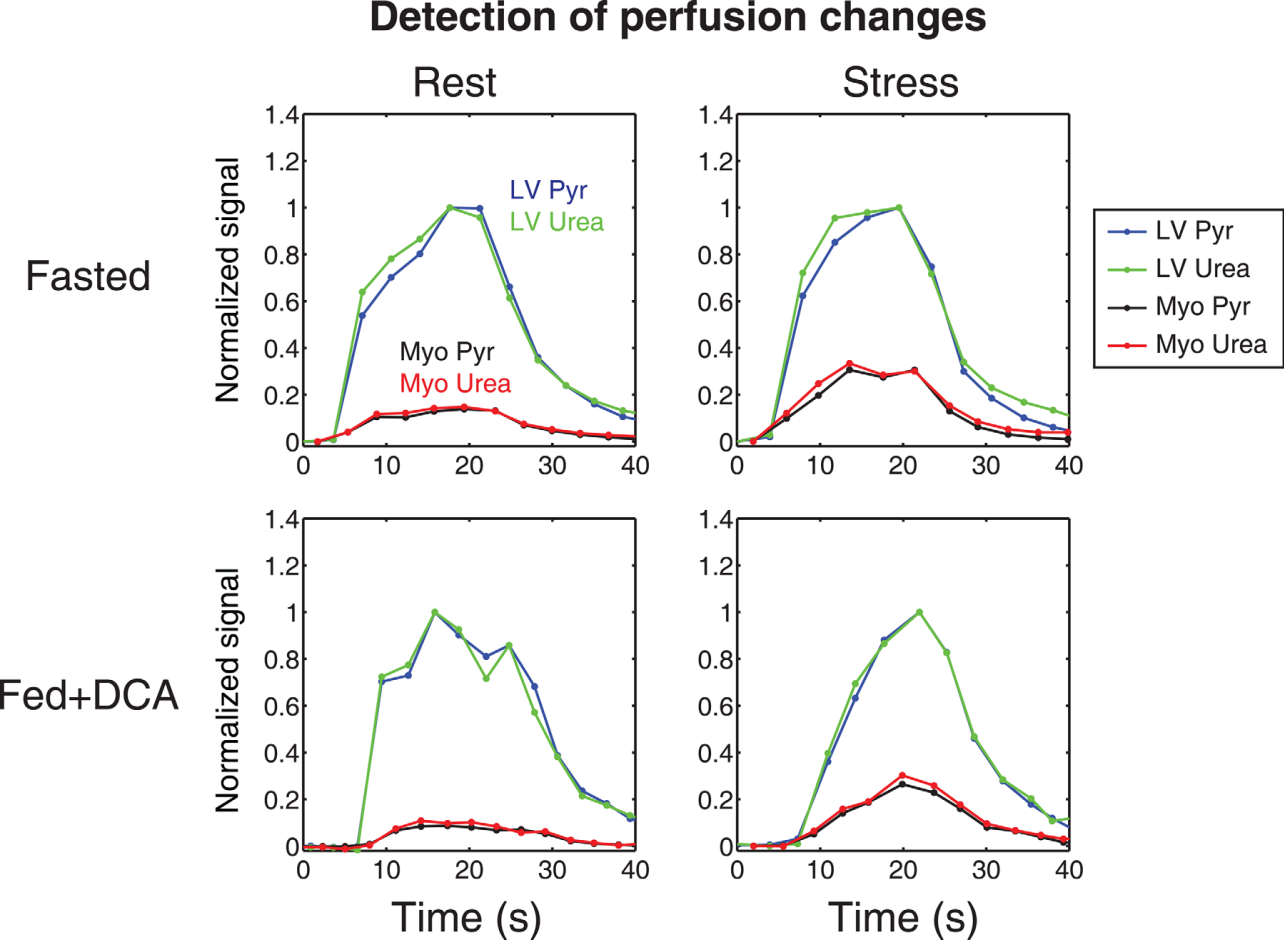
Time courses showing arrival of copolarized pyruvate and urea in the LV and subsequent arrival in the myocardial tissue in each combination of perfusion and metabolic state. An increase in perfusion during adenosine stress was revealed by the flow‐sensitized myocardial pyruvate and urea signals. The signals were normalized to the maximum LV pyruvate or urea signal as appropriate.

Figure 4 shows representative time courses of hyperpolarized [1‐^13^C]pyruvate and ^13^C‐urea as they pass through the LV and arrive at the myocardial tissue. Here, it is apparent that infusion of adenosine resulted in increased myocardial pyruvate and urea signals. The myocardial pyruvate and urea signals were not affected by fasting or feeding.

**Figure 5:**
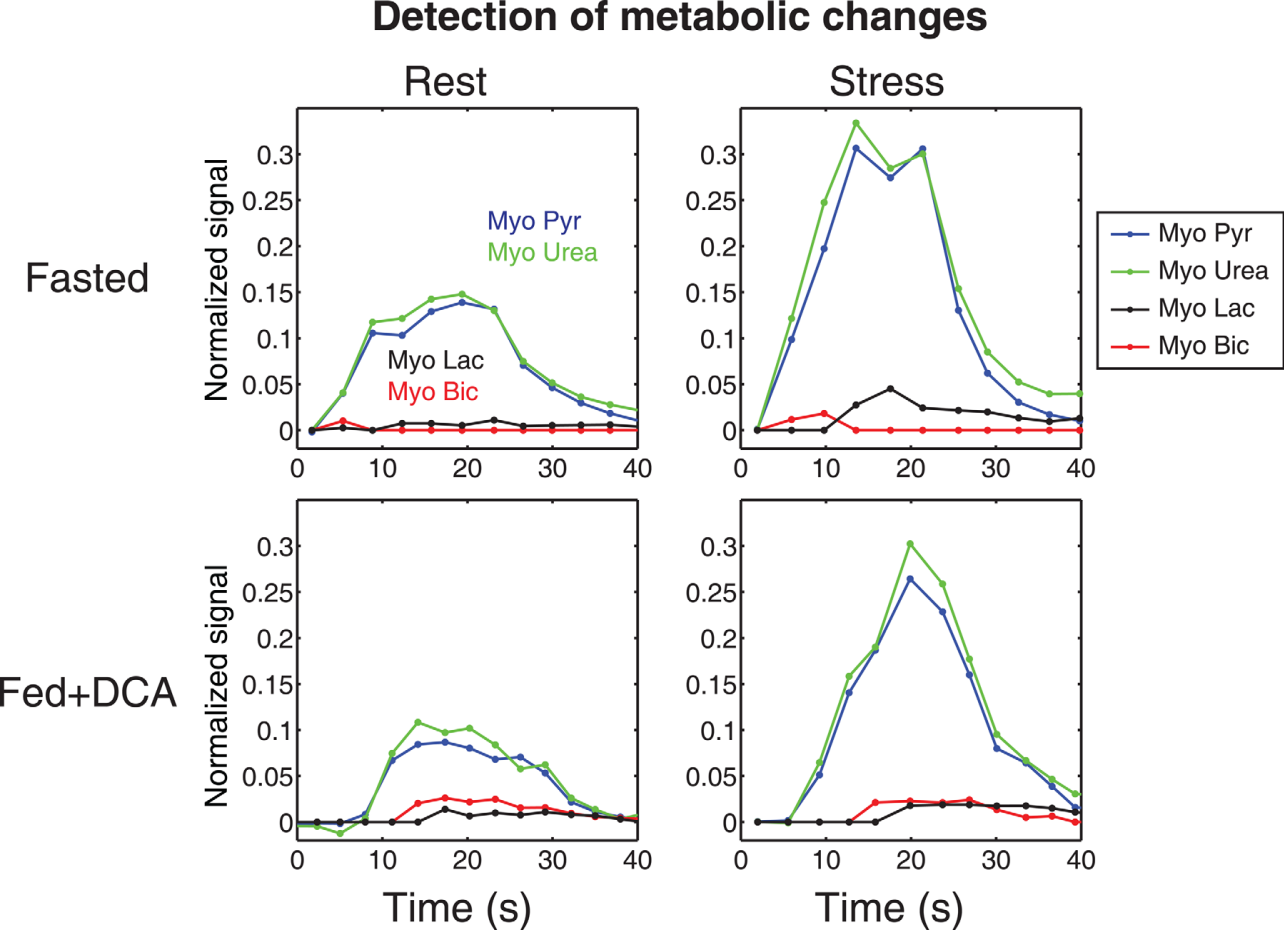
Time courses showing conversion of hyperpolarized myocardial pyruvate into bicarbonate and lactate in each combination of perfusion and metabolic state. A difference in myocardial bicarbonate production (in red) was observed between overnight fasted and fed with DCA injection states. The signals were normalized to the maximum LV pyruvate or urea signal as appropriate.

Figure 5 shows the conversion of hyperpolarized [1‐^13^C]pyruvate into ^13^C‐bicarbonate and [1‐^13^C]lactate in the myocardium. Here, there was an increase in myocardial bicarbonate in the fed state. The perfusion state (rest versus stress) did not affect the amount of bicarbonate observed, which is consistent with saturation of cardiac PDH following injection of 80 mM pyruvate 25.

**Figure 6:**
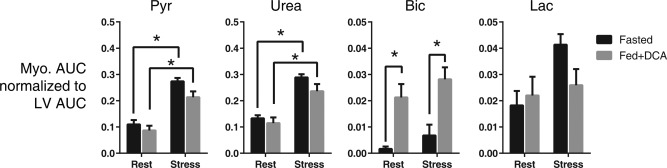
Semiquantitative measures of myocardial perfusion and metabolism using copolarized ^13^C pyruvate and urea. The AUC ratio between myocardial tissue and the LV is shown for each metabolite (pyruvate, urea, bicarbonate, and lactate). Two‐way ANOVA revealed significant differences in myocardial pyruvate and urea signals between perfusion states independent of the metabolic state of the animal. Significant differences in bicarbonate production were detected between metabolic states independent of the perfusion state of the animal. No significant differences in lactate signals were detected between each state.

Figure 6 shows a statistical comparison between myocardial pyruvate, urea, bicarbonate, and lactate signals obtained in each state. Two‐way ANOVA revealed a significant difference between rest and adenosine stress‐induced hyperemia for pyruvate and urea. A significant difference in myocardial bicarbonate production was also detected between animals that were fasted overnight and those fed via DCA injection. No significant differences were detected in myocardial lactate production following modulation of either metabolism or perfusion. No significant interaction terms were detected for each metabolite, suggesting that metabolic and perfusion alterations can be made and detected independently of each other. A statistical difference between myocardial pyruvate and urea levels was not detected in the resting state or the adenosine stress state.

## DISCUSSION

Rapid assessment of myocardial viability following acute myocardial infarction guides the clinical decision to proceed to revascularization. Clinical evidence shows that revascularization only results in improved outcomes if targeted to the presence of significant myocardial ischemia 2, 26, 27. A hyperpolarized MRI test, which directly grades the extent of myocardial ischemic damage by simultaneously assessing both blood flow to the tissue (perfusion) and cellular viability (intact metabolism), will thus have great clinical importance. This test is rapid and can be used in the acute setting to quickly determine the presence or absence of viable tissue.

In this study, we have demonstrated the feasibility of simultaneously assessing both perfusion and metabolism in the heart, by imaging a copolarized bolus of hyperpolarized [1‐^13^C]pyruvate and ^13^C‐urea. This builds upon previous work in which hyperpolarized ^13^C‐urea was used as a rapid probe of myocardial perfusion in the rodent heart 14. Hyperpolarized ^13^C images were shown to be sensitive to independent changes in metabolism and perfusion. Metabolic alterations via increased PDH flux between fasted and fed states led to increased ^13^C‐bicarbonate signal. Similarly, perfusion alterations via adenosine stress‐induced hyperemia led to increases in tissue [1‐^13^C]pyruvate and ^13^C‐urea signal.

The similarity between the shapes of the pyruvate and urea time courses may be surprising, given that urea is functionally inert, whereas pyruvate is metabolized in vivo and the ^13^C label is transferred to different metabolites. It is likely that this is because uptake and conversion represents a small fraction of the injected ^13^C pyruvate dose, which is supported by the small bicarbonate‐to‐pyruvate and lactate‐to‐pyruvate ratios (<5%) shown in Figure 6. This situation may be altered in ischemia, where hypoxia leads to increased anaerobic glycolysis and increased lactate levels within the heart. Furthermore, the use of hyperpolarized ^13^C‐urea as a perfusion agent is expected to become important in diseased states in which pyruvate conversion (ie, PDH flux) is low. Optimized acquisitions that do not disturb the reservoir of hyperpolarized [1‐^13^C]pyruvate magnetization, while simultaneously providing perfusion information via the use of hyperpolarized ^13^C‐urea, will likely improve sensitivity to inherently low ^13^C‐bicarbonate levels.

The readout duration (15 ms) used in this study is fairly long, considering typical line widths of 40 Hz for pyruvate and urea in the heart at 7 T. This long readout duration results in blurring in the spiral images due to B_0_ inhomogeneity. However, the fitted line width for bicarbonate, which is produced intracellularly, is 15 Hz, which reflects the more restricted environment the metabolite is found in. Thus, the bicarbonate images are typically less affected by B_0_ inhomogeneity. The effects of the long readout duration can be reduced by improving the B_0_ shim over the heart using a single‐voxel shimming method, or by better postprocessing using a separately acquired ^1^H field map 28, 29.

The temporal resolution of multiecho acquisitions is determined by the number of echoes required to accurately reconstruct the full set of metabolite images. Here, nine RR intervals (∼1.5 s) are required to obtain pyruvate, urea, and downstream metabolite images in a single slice. Several strategies are available to improve the temporal resolution of this scan. The oversampled k‐t spiral approach requires fewer excitations by effectively obtaining data in a spectroscopic fashion 29. This strategy requires a higher slew rate than is available in the gradient set used here, but has recently been shown to allow single‐shot imaging of ^13^C pyruvate and its downstream metabolites.

Another approach would be to restrict the number of excited metabolites by spectrally selective excitation 30, 31, 32, 33, 34, 35. An ECG‐gated three‐dimensional EPI approach using spectral‐spatial excitation provides entire rodent cardiac volumes corresponding to single metabolites. It is important to note that the close chemical shifts of ^13^C‐urea and ^13^C‐bicarbonate (∼3 ppm = 200 Hz at 7 T), coupled with difficult shimming conditions in the heart, make it very challenging to implement spectral‐spatial excitation of single NMR lines. Instead, a multiband RF pulse 28, 36 can be used to excite different metabolites with different flip angles. In these designs, the pyruvate peak can be placed within a minimum in the spectral profile to minimize the usage of this pool, while the flip angle delivered to urea can be large, as the magnetization usage does not matter. The flip angle for the downstream metabolites can also be increased to improve the signal‐to‐noise ratio of these images. This strategy can be beneficial in pathophysiological states in which PDH flux is decreased and bicarbonate signal approaches the limit of detection.

It is of interest to consider the feasibility of this method for studies at the clinical field strength of 3 T. The main issues are those of shimming and gradient performance. At 3 T, whole heart line widths of 1 ppm (∼30 Hz for ^13^C) are achieved 31. Interleaved spectral‐spatial excitation with single‐shot spiral excitation has been shown to adequately resolve [1‐^13^C]pyruvate and its downstream metabolites in the porcine heart, using RF pulse durations of 15 ms. The challenge with spectrally selective excitation is the relatively close chemical shift at 3T (Δ ≈ 75 Hz) of ^13^C‐bicarbonate (160 ppm) and the additional ^13^C‐urea resonance (162.5 ppm). A spectral‐spatial RF pulse that excites either urea or bicarbonate constrains the pulse duration to 2/Δf = 27 ms. Alternatively, the IDEAL approach used in this study can be used, either with or without spectral‐spatial excitation. If spectrally selective excitation is used, bicarbonate and urea can be excited together while leaving the pyruvate resonance untouched. A low number of echoes (∼3) can then be used to resolve these two metabolites.

Regarding gradient performance and the feasibility of using flow sensitizing gradients at clinical field strength, it is important to note the contributing factors to spoiling efficiency. Typical gradient performance on clinical 3 T scanners are approximately 25% that of the gradient set used in this study (G_max_ ∼ 40 mT/m versus 170 mT/m). At 3 T, the longer signal persistence allows the readout duration and echo time to be increased by approximately a factor of 2 given similar shimming conditions. The first gradient moment (*m*_1_ = ∫ *G*(*t*)*t*d*t*) of a bipolar gradient scales with T^2^, where T is the duration of the gradient, so given these constraints similar performance can be achieved at 3T.

## CONCLUSIONS

It is feasible to simultaneously image the first passage of a bolus of copolarized ^13^C‐pyruvate and ^13^C‐urea through the rodent heart, and the subsequent uptake and metabolic conversion can be quantitatively evaluated. Increases in myocardial perfusion relative to rest can be detected during adenosine stress‐induced hyperemia, and independent increases in myocardial metabolism can be detected between different metabolic states, in a single scan. This probe of both myocardial perfusion and metabolism is anticipated to enable metabolic study of the heart in acute scenarios.
